# Characterization of Visual Percepts Evoked by Noninvasive Stimulation of the Human Posterior Parietal Cortex

**DOI:** 10.1371/journal.pone.0027204

**Published:** 2011-11-08

**Authors:** Peter J. Fried, Seth Elkin-Frankston, Richard Jarrett Rushmore, Claus C. Hilgetag, Antoni Valero-Cabre

**Affiliations:** 1 Department of Anatomy and Neurobiology, Boston University School of Medicine, Boston, Massachusetts, United States of America; 2 Department of Computational Neuroscience, University Medical Center Hamburg Eppendorf, Hamburg, Germany; 3 Institut du Cerveau et la Möelle, Université Pierre et Marie Curie, Paris, France; National Institute of Mental Health, United States of America

## Abstract

Phosphenes are commonly evoked by transcranial magnetic stimulation (TMS) to study the functional organization, connectivity, and excitability of the human visual brain. For years, phosphenes have been documented only from stimulating early visual areas (V1–V3) and a handful of specialized visual regions (V4, V5/MT+) in occipital cortex. Recently, phosphenes were reported after applying TMS to a region of posterior parietal cortex involved in the top-down modulation of visuo-spatial processing. In the present study, we systematically characterized parietal phosphenes to determine if they are generated directly by local mechanisms or emerge through indirect activation of other visual areas. Using technology developed in-house to record the subjective features of phosphenes, we found no systematic differences in the size, shape, location, or frame-of-reference of parietal phosphenes when compared to their occipital counterparts. In a second experiment, discrete deactivation by 1 Hz repetitive TMS yielded a double dissociation: phosphene thresholds increased at the deactivated site without producing a corresponding change at the non-deactivated location. Overall, the commonalities of parietal and occipital phosphenes, and our ability to independently modulate their excitability thresholds, lead us to conclude that they share a common neural basis that is separate from either of the stimulated regions.

## Introduction

Phosphenes are brief visual percepts caused by mechanically or electrically induced depolarization of cells in the retina or visual brain [1]. Cortically evoked phosphenes were first elicited in humans by applying alternating electrical currents through scalp electrodes [2]. More recently, transcranial magnetic stimulation (TMS) has been used to safely, painlessly and noninvasively evoke phosphenes in the human occipital cortex [3]. TMS works on the principles of electromagnetic induction [4]: current is passed briefly through a coil held against the scalp, generating a rapidly changing electromagnetic field; this noninvasive field induces a brief focal electrical current in the underlying cortex, which in turn produces a synchronous depolarization of neurons in the target region (for a review, see [5]).

TMS-evoked phosphenes are a straightforward means to map the functional organization of visual cortical areas in both intact humans [6], [7] and those with blindness or blindsight [8], [9]. In addition, phosphenes have been widely used to characterize cortico-cortical interactions underlying visual awareness and visuo-spatial attention [10]–[14]. More recently, phosphenes have proved instrumental in demonstrating the state-dependent nature of neurostimulation methods [15]–[17].

Phosphenes are also used to assess the relative excitability of visual cortex [18], [19]. By convention, this measure is quantified as the phosphene threshold, and corresponds to the magnetic field intensity (measured as a % of the maximum stimulator output) that elicits a positive report of a perceived phosphene in approximately 50% of pulses. Under normal conditions, the phosphene threshold is a very stable measure of excitability that can be followed longitudinally across time or experimental conditions in both healthy individuals and patients [20]–[22]. Consequently, phosphene thresholds have been widely used to assess the outcome of rTMS and other neuromodulation regimes [23], [24], monitor cortical plasticity induced by light deprivation [25]–[27], and increase our understanding of certain neurological disorders, such as migraine [28]–[32].

Phosphenes have traditionally been studied in early visual areas (V1–V3) of occipital cortex, where they tend to appear as small stationary blobs or shapes (wedges, crescents, ellipses, etc.) [6]. Regardless of appearance, all occipital TMS phosphenes share three essential characteristics: they are perceived regardless of whether the participant's eyes are open or closed; they appear contralateral to the stimulated hemisphere; and their perceived position in visual space predictably changes with the location of fixation [3], [7]. Changing the coil position also alters phosphene location, reflecting the point-to-point retinotopic organization of early visual areas [3], [6], [7]. In later visual areas (i.e., V4, V5/MT+), where neurons have broader receptive fields and process specific features, TMS produces phosphenes that tend to be larger, display a coarser retinotopic organization, and even adopt qualities such as motion, texture or color [3], [8], [33], [34]. Together, these observations suggest that the characteristics of phosphenes are closely related to the function and receptive field organization of the stimulated neurons.

Recently, Marzi et al. [35] published the first documentation of phosphene-like percepts resulting from parietal TMS. In the context of investigating parietal/occipital differences in interhemispheric transfer time, the authors reported that stimulation of the left and right intraparietal sulcus (IPS) evoked visual percepts that were similar to occipital phosphenes in many respects. While this study was instrumental in establishing the existence of parietal phosphenes, the authors did not perform a more systematic characterization of their features. Thus it remains unclear whether parietal phosphenes have properties that reflect the intrinsic function of local IPS neurons.

The IPS is a region of visual association cortex located fairly rostrally along the dorsal visual stream. As such, it receives visual input through the slow retino-geniculo-striate pathway, via extastriate areas, as well as the fast retino-tectal pathway, via the pulvinar and other thalamic nuclei [36]–[38]. Within the IPS region, neurons are organized into rough visual maps representing the contralateral hemifield [39], [40]. These neurons have larger receptive fields than in early visual areas [41], [42] and are thought to incorporate a frame-of-reference representing egocentric or global rather than retinocentric space [43]–[45]. On the basis of these regional differences in neuronal properties, we hypothesized that phosphenes induced by TMS of the IPS would display distinct features from those evoked in early visual areas. Specifically, we predicted that parietal phosphenes would appear larger than occipital phosphenes and less anchored to eye movements.

In addition to receiving feed-forward visual input, the IPS has feedback projections onto multiple cortical and subcortical visual structures [46], [47]. Through these connections, the IPS exerts a strong top-down modulatory effect on the activity in early (V1–V3) and later (V4, V5/MT+) visual areas [10], [14], [48], [49]. Given the functional connectivity between the IPS region and early visual areas, it is reasonable to presume that parietal and occipital phosphenes might depend in some way on activity in the corresponding region. If this is the case, then altering the excitability of one region should produce a corresponding change in the phosphene threshold measured at the other site.

The broad goal of the present study was to determine whether parietal phosphenes are generated directly by local mechanisms or emerge through indirect activation of other visual areas. To accomplish this goal, we directly compared phosphenes evoked in two discrete regions of visual cortex: the intraparietal sulcus in right posterior parietal cortex and early visual areas of the right occipital pole. In the first of two experiments, we used a custom-made documentation system, developed in-house to electronically record the subjective features of phosphenes evoked as participants fixated in different locations. In the second experiment, we examined the interdependence of parietal and occipital phosphenes using low frequency (1 Hz) repetitive TMS (rTMS) to temporarily deactivate each site in separate sessions [50], [51]. This combined approach allowed us to systematically characterize parietal phosphenes and elucidate their neural basis.

## Results

All participants (n = 23) consistently reported phosphenes following single pulse TMS to early visual areas in right occipital cortex and the intraparietal sulcus region in right posterior parietal cortex. At both locations, phosphenes conformed to inclusionary criteria: they appeared in the side of visual space contralateral to the stimulated hemisphere and were perceived regardless of whether participants' eyes were opened or closed. Importantly, phosphenes were not systematically reported following sham stimulation of phosphene regions or real TMS of non-phosphene regions.

### Results from Experiment 1

**Figure 1:**
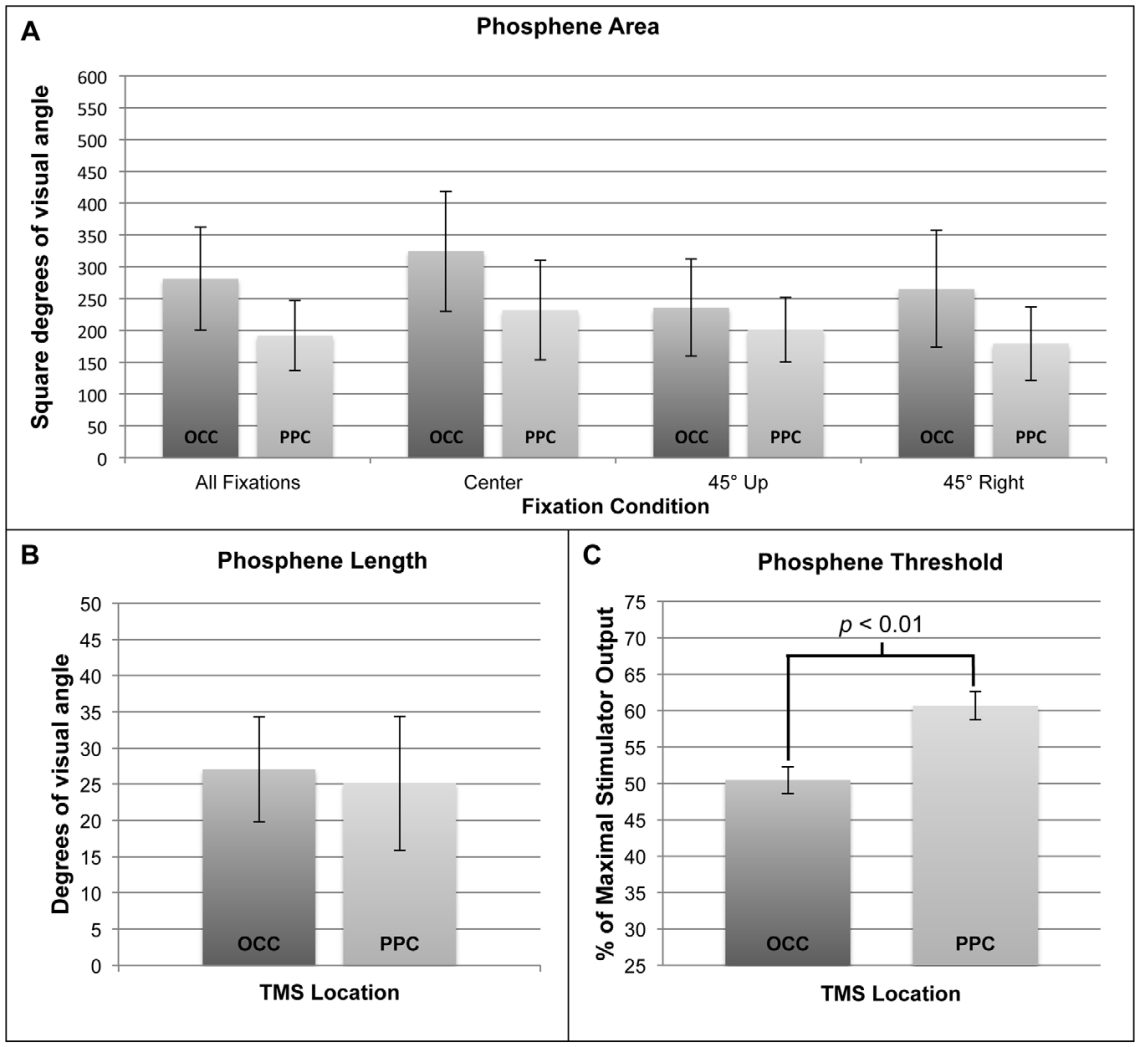
Results of Experiment 1: comparison of phosphene size and threshold. Phosphenes were reliably elicited in all participants (n = 9) at both occipital (OCC) and posterior parietal (PPC) locations. **A.** With stimulation intensity set to 110% of phosphene threshold, there were no significant differences (all *p*'s>0.1) in area with regard to either the location of TMS (OCC, PPC) or the direction of fixation (center, 45° up, 45° right). **B.** For the subset of phosphenes that were perceived (and drawn) as lines rather than enclosed shapes, there was no significant difference (*p*>0.5) with regard to the location of TMS (OCC, PPC). **C.** There were significantly higher stimulation thresholds for parietal phosphenes than their occipital counterparts (*p*<0.01). Error bars represent SEM.

As illustrated in Figure 1C, the two-tailed paired-samples t-test for phosphene thresholds (mean ± SEM: % of maximum stimulator output) demonstrated it took significantly greater intensity to consistently elicit parietal (60.7±2) than occipital phosphenes (50.4±2), *t*(8) = −3.71, *p*<0.01.

Considering phosphene size, the 2 (TMS site)×3 (fixation location) ANOVA for area (mean ± SEM: square degrees of visual angle), indicated no significant main effects of the phosphene site or the fixation location and no significant interaction, all *F's*<1.2, all *p's*>0.1. As depicted in Figure 1A, planned pairwise comparisons using two-tailed paired-samples t-tests revealed no significant difference between the area of occipital (281.4±81) and parietal phosphenes (192.1±55), *t*(8) = 1.025, *p*>0.1. Figure 1B illustrates that for the subset of phosphenes perceived as straight lines (mean ± SEM: degrees of visual angle), two-tailed paired-samples t-test showed no significant difference in the length of occipital (27.1±7) and parietal phosphenes (25.1±9), *t*(6) = 0.17, *p*>0.5. In sum, while occipital phosphenes tended to be larger than parietal ones, no significant group-level differences were observed with regard to either measure of phosphene size, area or length.

**Figure 2:**
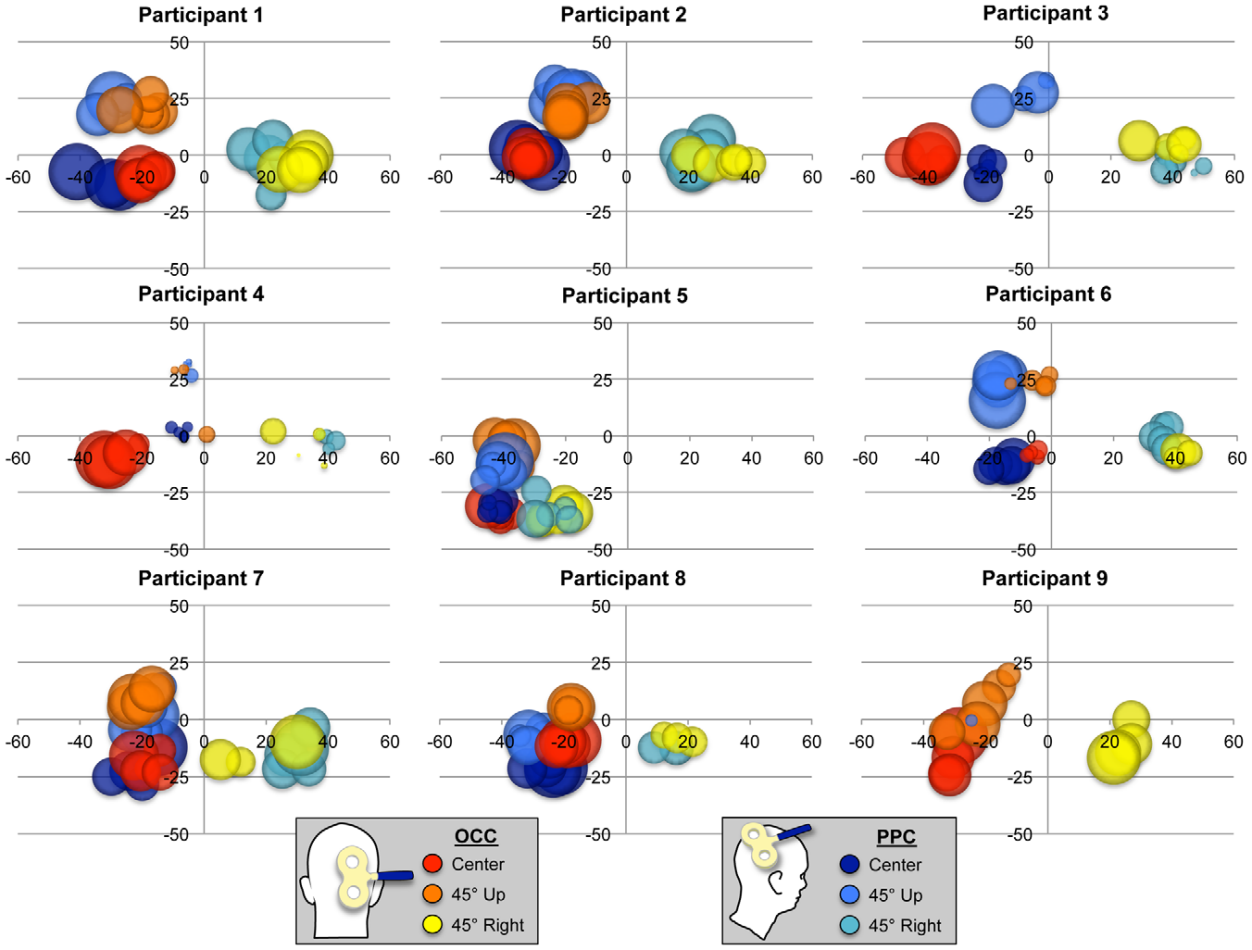
Experiment 1: Laser Tracking and Painting (LTaP) data. Each graph represents LTaP data from a single participant. Bubbles represent phosphene size (area of bubble = phosphene area) and position (center of bubble = phosphene center-of-gravity) for all six conditions: two stimulation sites (OCC, PPC)×three fixations (center, 45° up, 45° right). Axes are in degrees of visual angle.

Finally, a qualitative examination of drawings made by participants using the Laser Tracking and Painting (LTaP) system [52] revealed that parietal phosphenes, like their occipital counterparts, were perceived in the visual field contralateral to the TMS location, typically below the horizontal meridian. Moreover, as illustrated in Figure 2, this position reliably and predictably changed according to the location of fixation and indicates parietal phosphenes are perceived in a retinocentric frame-of-reference. A number of different shapes were depicted including wedges, ellipses, circles, bars, and lines. These varied, both within and across participants, but no obvious differences in geometry were found between the two stimulation sites. When prompted to compare the two, participants tended to report that parietal phosphenes appeared less vivid than and not as sharply demarcated as occipital ones.

### Results from Experiment 2

The 2 (rTMS target)×2 (phosphene site)×3 (time) repeated measures ANOVA revealed a significant main effect for site of phosphene induction (TMS), *F*(1,19) = 34.851, *p*<0.001; and time of stimulation (Time), *F*(1.7,31.7) = 4.586, *p*<0.05. These results demonstrate that the intensity required to elicit phosphenes (i.e., the threshold) was different for occipital and parietal stimulation and also varied with time (baseline, post-rTMS and recovery). In addition, the ANOVA revealed a significant interaction between the rTMS target and phosphene site, *F*(1,19) = 8.642, *p*<0.01) and between rTMS target, phosphene site, and time, *F*(2,38) = 19.729, *p*<0.001. This suggests that 1 Hz rTMS produces discrete and temporary changes in phosphene thresholds relative to the site of stimulation.

**Figure 3:**
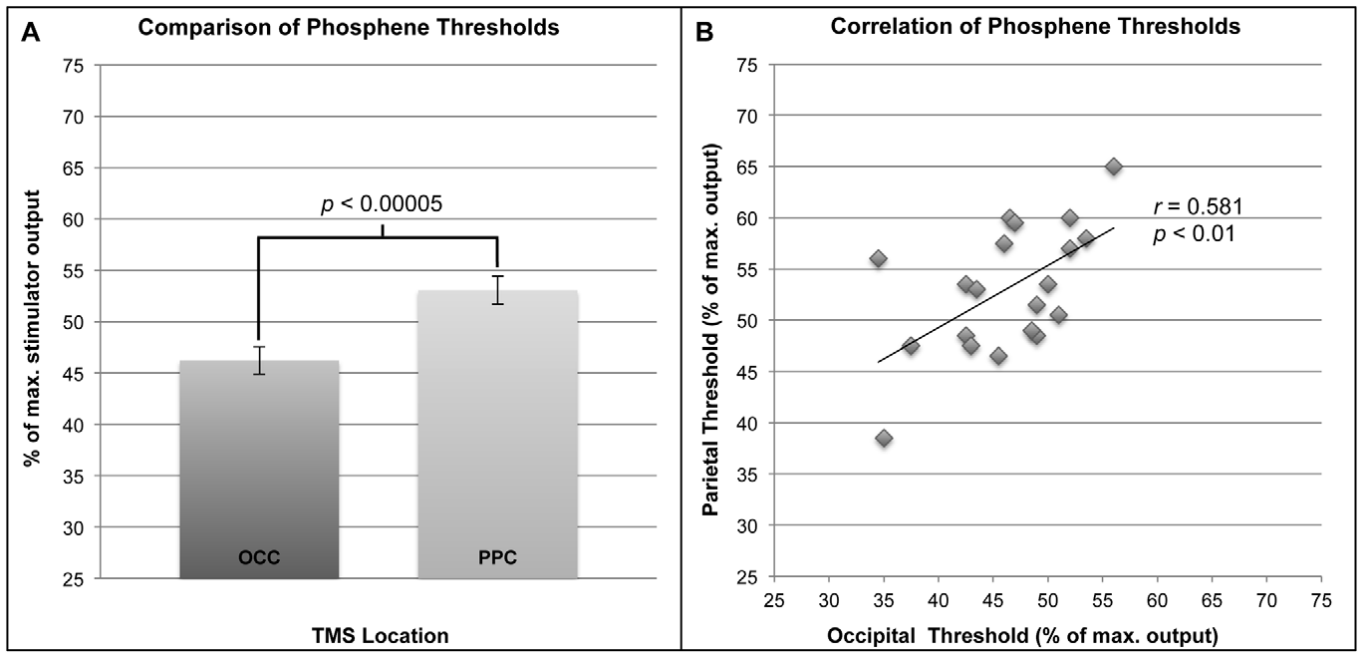
Results of Experiment 2: relationship of occipital and parietal phosphene thresholds. **A.** As with Experiment 1, phosphenes elicited from posterior parietal cortex (PPC) had higher thresholds than those from occipital cortex (OCC; *P*<0.00005). **B.** There was a significant positive correlation (*r* = 0.581) between thresholds of PPC and OCC phosphenes (*P*<0.01). Error bars represent SEM.

**Figure 4:**
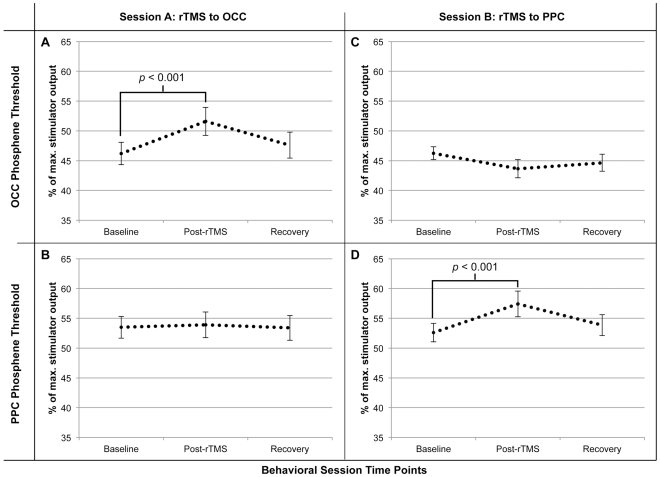
Results of Experiment 2: outcome of 1 Hz rTMS neuromodulation. **A–B.** In session A, suppressing excitability in right occipital cortex (OCC) with 1 Hz rTMS produced a significant increase in the OCC phosphene threshold (*p*<0.001), but did not change the phosphene threshold (*p*>0.1) assessed in right posterior parietal cortex (PPC). When re-assessed one hour later, thresholds at both sites were statistically unchanged from baseline (*p*'s>0.1). **C–D.** In session B, 1 Hz rTMS of PPC produced a similar increase in phosphene thresholds observed at that site (*p*<0.001), while OCC thresholds were statistically unchanged from baseline (*p*>0.01). When re-assessed one hour later, both OCC and PPC phosphene thresholds were statistically unchanged from baseline (*p*'s>0.01). Error bars represent SEM.

Figure 3A illustrates that, in accordance with Experiment 1, the two-tailed z-test revealed baseline phosphene thresholds (mean ± SEM: % of maximum stimulator output) were significantly higher for parietal (53.1±8) than occipital (46.2±7), *z*(38) = −4.17, *p*<0.001. As depicted in Figure 4C–D, post-hoc comparisons using two-tailed t-tests demonstrated that immediately after rTMS was applied to the parietal target, we observed a significant increase in the parietal phosphene threshold, *t*(19) = −3.89, *p*<0.001; as well as a slight decrease in the occipital threshold, which was not significant after correcting for multiple comparisons, *t*(19) = 2.27, *p*>0.01. By comparison, Figure 4A–B illustrates that immediately after rTMS was applied to the occipital target, we found a significant increase in the occipital phosphene threshold, *t*(19) = −4.11, *p*<0.001, and no change for parietal threshold, t(19) = −0.3, *p*>0.1. Regardless of the rTMS condition and site of induction, all thresholds measured 60 minutes after rTMS were not statistically different from baseline, all *t's*<2.3, all *p's*>0.01. Finally, as depicted in Figure 3B, a Pearson's correlation test indicated that occipital and parietal thresholds were strongly correlated with each other, *r*(20) = 0.581, *p*<0.01, indicating the response of these regions to TMS is governed by related factors. On the other hand, neither occipital nor parietal phosphene thresholds were correlated with motor thresholds, all *r's*<0.24, all *p's*>0.1, reflecting differences in the response to TMS between motor and visual cortices.

These results confirmed our findings from Experiment 1 of higher thresholds for parietal than occipital phosphenes. Moreover, the results from Experiment 2 demonstrated that reducing excitability at either cortical location exclusively elevated the threshold from the site receiving rTMS, but did not significantly modulate phosphene thresholds elicited from the non-stimulated site. Thus, in spite of the similarities of occipital and parietal percepts documented in Experiment 1, these results indicate that at both sites, local mechanisms contributing to phosphene generation are independent.

## Discussion

In accordance with the results of Marzi et al. [35], we found no systematic differences in size, shape or location between parietal and occipital phosphenes. In addition, phosphene location was just as dependent on gaze fixation for parietal as occipital phosphenes. Thus, phosphenes evoked by TMS of IPS were not found to exhibit the unique properties associated with neurons in that region and were no different in most measures from occipital phosphenes. However, parietal phosphenes did have a significantly higher threshold than occipital phosphenes. Anatomical studies have shown the distance between the scalp and cortical surface is greater for parietal than for occipital cortex [53], [54]. Therefore, higher parietal thresholds are likely due to physical rather than neural differences. This interpretation is reinforced by strong correlations between the phosphene thresholds in the two regions. Overall, these findings lead us to conclude that parietal and occipital phosphenes have similar qualities and likely share a common neural basis.

The driving hypothesis of this study was that phosphenes induced by TMS should reflect the intrinsic attributes of the neurons being stimulated [8], [7], [33], [34]. In contrast, phosphenes perceived following IPS stimulation had no distinct features from those evoked in occipital cortex. The functional organization of neurons in the IPS region has been well characterized in both humans and non-human primates; for reviews, see [55] and [40]. Notably, neurons in the IPS have larger receptive fields than in early visual areas [41], [42] and given the role of this region in the allocation of attention to extrapersonal space [56]–[60], IPS neurons are thought to incorporate a frame-of-reference representing egocentric or global rather than retinocentric space [43]–[45], [61]. Based on these properties, IPS phosphenes were predicted to be larger and less anchored to eye movements than those evoked from stimulation of early visual areas. Instead, phosphenes from both sites were of equivalent size and similarly changed location with fixation. Thus parietal phosphenes appear to reflect features attributed to early visual areas, rather than those associated exclusively with the IPS region.

These results cast doubt on the neural basis of parietal phosphenes. It has been suggested that phosphenes might be generated by stimulation of the optic radiations [3], [7]. However, given the close anatomical relationship of the optic radiations to the lateral ventricles [62], it is unlikely that parietal TMS (even at suprathreshold intensities) could penetrate deep enough to reach them. The most parsimonious explanation for the current findings is that stimulation of the IPS indirectly activates early visual areas. Connections between the IPS region and early visual areas consist of both feed-forward visual input as well as feedback projections [36], [63]. In particular, the latter pathways facilitate the robust top-down influence of the IPS region on early visual areas [10], [14], [48], [49], Therefore, it is feasible that TMS induced depolarization of IPS could propagate (either antidromically or orthodromically) to early visual areas, where the awareness of the percept could occur.

To investigate this hypothesis, the parietal phosphene threshold was measured before and immediately after deactivating the occipital phosphene site with 1 Hz rTMS. If the generation of parietal phosphenes depends on activity in early visual areas, suppression of occipital excitability should alter the parietal phosphene threshold. In contrast, the results show that deactivation of the occipital phosphene site temporarily increased the phosphene threshold at that location, but did not change the threshold for parietal phosphenes. This indicates that parietal phosphenes are not dependent on activity in the occipital phosphene site. The inverse of this relationship produced the opposite results: applying 1 Hz rTMS to the IPS region increased the threshold for parietal, but not occipital phosphenes. Overall, these results demonstrate that parietal and occipital phosphenes are functionally independent from each other, and that the source of parietal phosphenes is not the region of early visual cortex we targeted for occipital phosphenes.

A reconciliation of the commonalities of parietal and occipital phosphenes with their functional independence is possible if neither of the stimulated areas generates the perception of the phosphene. In this view, both the occipital and parietal phosphene sites represent independent nodes that, when stimulated by suprathreshold TMS, direct the activation of a common third region or network on which the two systems might converge. Thus each phosphene is the product of activity of local circuits as well as neurons in the third region. The former govern factors such as the excitability threshold and can be independently modulated by rTMS, while the latter confer features upon phosphenes based on intrinsic neural properties and are not susceptible to the effects of remotely applied rTMS.

In support of such a hypothesis, Taylor et al. [64] evoked phosphenes in the occipital pole while simultaneously recording the EEG of participants. With stimulation intensity set to the 50% occurrence threshold, participants were asked to respond whether or not they perceived a phosphene. The only difference in EEG evoked activity between positive and negative phosphene reports occurred relatively late (160–200 ms) after delivery of the TMS pulse. This implication of this study is that conscious perception of a phosphene only occurs after substantial processing of neural activity induced by the TMS pulse. Therefore, if the awareness of a phosphene and its associated TMS induced depolarization are decoupled in time, it is reasonable to presume they also occur in different brain regions.

The region most likely to serve as the source of phosphene awareness is the primary visual cortex (V1). Lesions of V1 (or its input) result in the loss of conscious perception of incoming visual stimuli. Even in the absence of V1, some retinal input makes its way to higher visual areas (e.g., V5/MT+, IPS) via the tecto-pulvinar pathway, however this information does not reach the level of awareness, hence the term blindsight [65]. The same is true for TMS-induced activity: several studies [9], [12], [66] of blind, blindsighted, and intact individuals have shown V1 activity is necessary for the awareness of phosphenes evoked by stimulating V5/MT+. In the present study, the fact that there was no change in the ability to induce parietal phosphenes following deactivation of the occipital phosphene site would suggest that the latter did not correspond to V1. The scalp locations used to target occipital stimulation in this and similar studies [35], [67] lie at least 2 cm laterally from the midline. Given the retinotopic position of the phosphenes in the lower contralateral visual field, it is probable that the associated neural representation of V1 is in fact within the calcarine sulcus on the medial surface of the brain and thus poorly accessible to the currents induced by focal TMS. As such, it is more likely that occipital stimulation targeted some combination of V2 and V3 and activated projections to V1.

In sum, this study provides confirmation that stimulation of a region near the intraparietal sulcus in posterior parietal cortex reliably produces genuine phosphenes. Rather than adopting features associated with the intrinsic attributes of IPS neurons, these percepts appear similar to those evoked from stimulating visual areas V2–V3 in occipital cortex. Despite these commonalities, deactivation of the occipital and parietal sites revealed their functional independence. Thus we conclude that the perception of IPS and V2–V3 phosphenes occurs not from local TMS-induced activity, but rather from indirect activation of a common third region. Given its preeminent role in visual awareness and functional connectivity with both of the targeted regions, the strongest and most compatible interpretation is that V1 is the locus of phosphene perception for both IPS and V2–V3 stimulation.

## Materials and Methods

The present study comprised two independent experiments. Experiment 1 consisted of a single session per participant and compared the characteristic features of phosphenes induced in posterior parietal cortex to those generated by stimulating the pole of the occipital cortex. Experiment 2 required two sessions per participant and explored the functional dependence of the same two locations by using 1 Hz rTMS to modulate phosphene thresholds.

### Ethics Statement

All forms and procedures used in the study received approval by the Institutional Review Board at Boston University School of Medicine. All participants provided written consent upon enrollment in the study, and were thoroughly screened for exclusion criteria with regards to the risks of TMS [68] prior to each experimental session. Participants were compensated for their time at the end of each experimental session.

### Participants

A total of 23 healthy adults (15 male, 8 female) of mean age 27.9 years (range = 21.8–45.5) with no known history of neurological disease participated in one or both experiments of the present study (see Table 1 for demographic information).

**Table 1:** Study Demographics.

	Gender	Age (y)	Experiments	Naïve^b^
Participant 1	M	27.0	1 & 2	N
Participant 2^a^	M	28.6	1 & 2	N
Participant 3	M	24.2	1 & 2	Y
Participant 4	M	25.3	1 & 2	Y
Participant 5	M	27.8	1	Y
Participant 6	F	26.3	1	Y
Participant 7	F	22.5	1 & 2	Y
Participant 8	M	32.3	1 & 2	Y
Participant 9	M	24.5	1	Y
Participant 10^a^	M	40.3	2	N
Participant 11^a^	M	38.2	2	N
Participant 12	F	37.9	2	N
Participant 13	F	29.6	2	N
Participant 14	M	24.6	2	N
Participant 15	M	24.7	2	Y
Participant 16	F	21.8	2	Y
Participant 17	F	23.8	2	Y
Participant 18	M	26.5	2	Y
Participant 19	M	22.5	2	Y
Participant 20	M	23.2	2	Y
Participant 21	M	45.5	2	Y
Participant 22	F	21.8	2	Y
Participant 23	F	22.8	2	Y

a Was a co-author of the study.

b No experience of TMS-induced phosphenes prior to the study.

### Transcranial Magnetic Stimulation

In both experiments, phosphenes and motor responses were induced by TMS using a Magstim 200 monophasic stimulator attached to a standard 70 mm diameter figure-of-eight coil for focal stimulation (Magstim Co. Ltd., Dyfeld, Wales, UK). The coil was held against the scalp with the center tangential to the site of stimulation and pulses were delivered either by stepping on a pneumatic foot switch or by pressing the trigger button on the front of the stimulator. Pulses were spaced at least 5–10 seconds apart (≤0.2 Hz); this rate served both to limit the temporal predictability of pulse onset and to avoid the lasting cumulative effects of rTMS [69]. Sham TMS was performed at the same locations by positioning the edge of the coil tangentially at 90° against the scalp, and discharging pulses at 80% of stimulator output, thus eliciting similar accompanying acoustic and somatosensory sensations, without inducing significant currents in the brain. Given the inherently subjective nature of phosphene perception, we randomly interposed catch trials, consisting of sham pulses delivered to phosphene sites, as well as real pulses delivered to non-phosphene areas (e.g., scalp vertex, primary motor cortex, etc.), to further assess participants' reliability in reporting the presence or absence of phosphenes.

In Experiment 2, rTMS was applied using an air-cooled 70 mm figure-of-eight focal coil (Magstim) attached to a Magstim SuperRapid biphasic stimulator. The coil was kept fixed in place for the duration of stimulation with the assistance of a multi-joint adjustable Magic Arm (Manfrotto, Italy). The sequence of pulses was programmed and initiated using Magstim Rapid Session Software (v 4.0). The pattern of stimulation consisted of one pulse per second (1 Hz) for 15 minutes (900 pulses total) at 90% of the local phosphene threshold value, which was determined separately from baseline threshold using the air-cooled coil and SuperRapid stimulator. Based on prior research [23], [70] and our own preliminary data, this rTMS pattern should result in a period of reduced excitability lasting at least 10 minutes, thus providing sufficient time to assess phosphene thresholds at both locations. During rTMS, participants were seated comfortably against a portable massage chair, with their face in a headrest, and were instructed to keep their eyes open as much as possible to avoid any confounding effects of light deprivation.

TMS was directed to scalp locations overlying three cortical sites: the pole of the right occipital cortex, corresponding most likely to early visual areas V2–V3, although stimulation of V1 cannot be ruled out [67]; borders of the IPS within the right posterior parietal cortex; and the region of primary motor cortex (M1) representing either the abductor pollicis brevis muscle (thumb) or first dorsal interosseous muscle (index finger). This last site served both to index the excitability of non-visual cortex for comparison with phosphene thresholds (Experiment 2) as well as to confirm the reliability of phosphene reports in an area that should not induce such percepts (Experiment 1 & 2). These three areas of stimulation were determined initially using anatomical skull landmarks and the “International 10–20 system” for EEG electrode placement. The position of the coil was then fine adjusted until a reliable behavioral response (i.e., an unambiguous phosphene report or a visual muscle twitch) was evoked. These sites were then marked on a snug-fitting Lycra™ swim cap worn by the participant. The approximate location (and orientation) of the TMS coil for each of these stimulation sites was as follows: 4–5 cm laterally from the vertex (EEG coordinate C_Z_) for M1 (handle pointing medial to lateral, away from C_Z_); 2 cm dorsally and 2 cm laterally from the inion for V2–V3 (handle pointing medial to lateral, away from the inion), and directly over EEG coordinate P_4_ for the IPS region (handle pointing ventromedial to dorsolateral, away from the inion). The use of EEG coordinates to guide TMS placement over functional brain areas represents an economical and practical tradeoff over more precise neuroimaging-based methods. With consequence for the present study, the relationship of P_4_ to the right IPS has been validated by several studies [71], [72]. All three of the aforementioned sites are common starting points when using TMS to measure cortico-spinal excitability from primary motor cortex [73], elicit retinotopically organized phosphenes from occipital cortex [67], or interact with visuo-spatial processes in posterior parietal cortex [14]. Similarly, these coil orientations have been shown to produce the optimal stimulation of the intended region, while minimizing stimulation of other significant cortical areas or musculature of the neck and face [19], [67], [74].

### Phosphenes and Motor Responses to TMS

We used the following established inclusion criteria for occipital phosphenes: a brief and spatially circumscribed visual percept, appearing in the left visual hemifield (contralateral to the stimulated hemisphere), immediately following real TMS only, and occurring regardless of whether the participant's eyes were opened or closed. The inclusion criteria for parietal phosphenes were the same as for occipital, although we would accept percepts that were less circumscribed and without restriction on visual field location, since there is some debate as to whether neurons in the human right IPS have bilateral or purely contralateral representations of visual space. A small number of potential participants (n = 3) were not included in the study on for the following reasons: an inability to perceive phosphenes in either of the two locations; reporting visual percepts following TMS that did not adhere to the aforementioned criteria or proved highly inconsistent; or reporting visual percepts following sham TMS to visual regions or real TMS to non-visual regions.

In both experiments we measured phosphene thresholds for the occipital and parietal sites. Additionally, in Experiment 2, we assessed the motor threshold at M1. Regardless of site, the same protocol was used at all times: with intensity initially set at 50–60% of the maximum stimulator output, we applied a series of at least 5 pulses at a maximum of 0.2 Hz and recorded whether each pulse produced a visible muscle twitch of the contralateral thumb or index finger (TMS to M1) or the unambiguous report of a qualified phosphene (TMS to V2–V3 and IPS). When assessing the occurrence of phosphenes, participants were instructed to respond verbally by stating “yes” if they definitely perceived a phosphene, “no” if they definitely did not perceive a phosphene, or “maybe” if they perceived something, but were unsure if it was a phosphene or some other sensation such as an eye blink. A “maybe” response was not counted as a positive or negative report, but instead initiated a follow-up pulse. Based on these responses, the stimulation intensity was subsequently adjusted using the following algorithm: if at least four consecutive pulses resulted in a “no” response, the stimulation intensity was increased by 10%; if at least four successive pulses resulted in a “yes” response, stimulation intensity was decreased by 10%; otherwise, if the series of pulses resulted in a mixture of “yes” and “no” responses, stimulation intensity was adjusted in smaller increments (1–5%). This process was repeated until reaching the minimum intensity value that resulted in positive reports from 50% of at least 10 delivered pulses. If no single intensity resulted in an occurrence of exactly 50%, we used the criteria of at least 40%, but no more than 60% positive reports. The advantage of this approach, which is similar to methods used in other phosphene studies [9], [11], [13], [14], [28], [29], is that it can be performed in approximately 2–4 minutes and thus works within the constraints imposed by the rapidly decaying effects of rTMS. Furthermore, a reliability study (data not shown) confirmed this method of assessing phosphene thresholds produces comparable results to more rigorous (and therefore longer) techniques based on randomized sampling [7], [67].

### Detailed Methods for Experiment 1

A subgroup of nine adults (seven males, two females) of mean age 26.4 years (range = 22.5–32.0) with no known history of neurological disease, including one of the co-authors of the study (PJF), participated in this experiment. Seven out of the nine participants were naïve to TMS-induced phosphenes, nonetheless they did not require significant training to consistently perceive and report them. The aim of this experiment was to characterize phosphenes evoked from applying TMS to the IPS region of posterior parietal cortex and compare their features (size, shape, location, and frame-of-reference) to those induced by stimulating early visual areas of the right occipital cortex.

**Figure 5:**
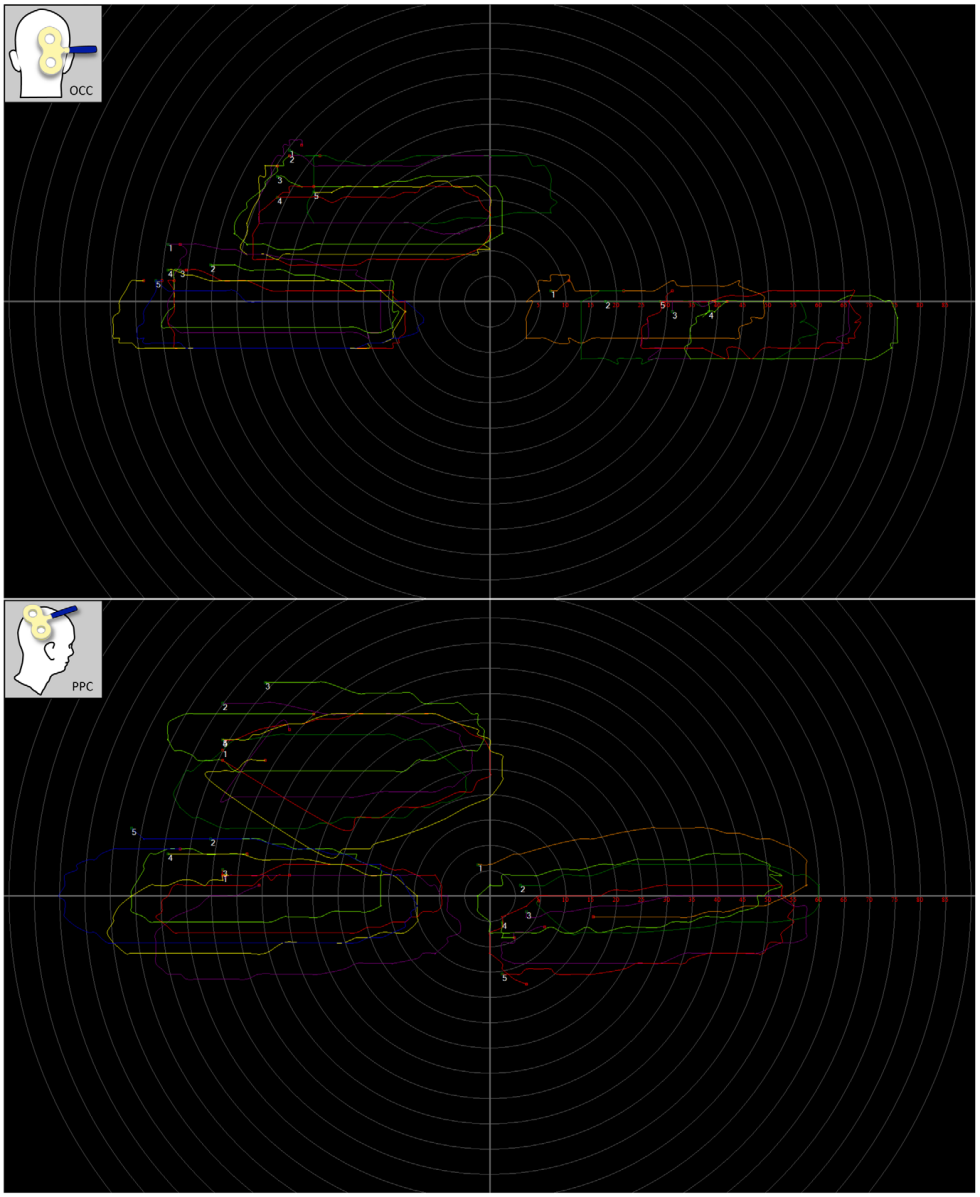
An example of output from the Laser Tracking and Painting (LTaP) system. **Upper panel.** LTaP output from one participant when TMS was applied to the pole of the right occipital cortex (OCC) during three fixation conditions (center, 45° up, 45° right). **Lower panel.** LTaP output from the same participant when TMS was applied to the right posterior parietal cortex (PPC) during the same three fixation conditions. Axes, numbers, and concentric circles were not visible on the screen during the experiment.

Experiment 1 consisted of a single session for each participant divided into two parts. In the first part, occipital and parietal phosphene sites were identified for each participant and marked on his or her swim cap. Using these locations, we determined the phosphene thresholds. Next, participants were seated in a dimly lit (∼0.5 cd/m^2^) room, at a distance of approximately 45 cm (measured from nasion) in front of a dual-sided projection screen (Da-View fast-fold® deluxe, Warsaw IN, USA) so that it filled their entire visual field, and given a modified green laser pointer (532 nm, 50 mW) that was held in their dominant hand. The participant was instructed to fixate on a point at the center of the projection screen while single pulses were delivered to occipital and parietal locations at 110% of the phosphene threshold recorded for each site. After each pulse, participants were asked whether they perceived a phosphene and if so, to trace the outline on the screen using the laser pointer. The Laser Tracking and Painting (LTaP) system (Laboratory of Cerebral Dynamics, Plasticity and Rehabilitation, Boston, MA, USA) documented the path of the laser pointer as X–Y coordinates and briefly displayed the outlined shape of the phosphene for near-instantaneous feedback (for a complete and detailed description of the LTaP system, see [52]). Once 5 phosphenes were successfully recorded in this manner, the process was repeated with the participant fixating on a point located 45° above, and then, 45° to the right of the center of the screen. As illustrated in Figure 5, a total of 30 phosphenes were collected for each participant: 5 phosphenes per fixation×3 locations of fixation (center, 45° up, 45° right) per stimulation site×2 stimulation sites (occipital and parietal).

We recorded the following data for each participant: two phosphene thresholds (measured as a % of maximal stimulator output) and 30 arrays of X–Y coordinates, each corresponding to the outline of a single perceived phosphene. Each X and Y value was transformed from a pixel value to a degree of visual angle using the following formula:
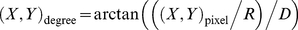
(1)Where (*X,Y*)_degree_ is the transformed value, (*X,Y*)_pixel_ is the native value obtained from the LTaP system, *R* is the ratio of the resolution output of the projector to the size of the projected image (12.5 pixels/cm) and *D* is the distance between the participant and the projection screen (45 cm). These transformed, standardized coordinates allowed us to calculate the area of phosphenes (in square degrees of visual angle). These data were averaged by participant, TMS site, and fixation location and entered into a 2 (TMS site: occipital and parietal)×3 (fixation location: center, 45° up, 45° right) repeated-measures analysis of variance (ANOVA) with a 95% confidence interval (α = 0.05). Mauchly's test indicated that the assumption of sphericity had been violated for the main effect of Fixation location, χ^2^(2) = 7.59, *p*<0.05), therefore degrees of freedom were corrected using Huynh-Feldt estimates of sphericity (ε = .6). For all other effects and interactions, the assumption of sphericity was met, all χ^2^'s<2, all *p*'s>0.1, allowing us to use uncorrected degrees of freedom. Pair-wise comparisons were computed using two-tailed paired-samples t-tests, with a more conservative 99% confidence interval (α = 0.01) to account for multiple comparisons. In addition, occipital and parietal phosphene thresholds were compared using a two-tailed paired-samples t-test.

A low number of phosphenes (12 occipital and 19 parietal) were perceived as straight lines rather than enclosed shapes, thus their extent could only be measured in terms of total length. These data were averaged for each participant independent of gaze direction due to the small number of samples and compared using a two-tailed independent-samples t-test. A small number of phosphenes (6 occipital and 1 parietal) were excluded from all analyses because a software error resulted in a truncated recording.

### Detailed Methods for Experiment 2

A subgroup of 20 adults (13 males, 7 females) of mean age 28.2 years (range = 21.8–45.5) with no known history of neurological disease, including three co-authors of the study (PJF, CCH and AV-C), participated in the experiment. An additional five had participated in the previous experiment, while nine were naïve to TMS-induced phosphenes. As with the previous experiment, participants required only minimal training to consistently perceive and report phosphenes. The aim of this experiment was to determine whether parietal and occipital phosphenes were independent and generated by local mechanisms, or interdependent, reflecting a common neural basis.

**Figure 6:**
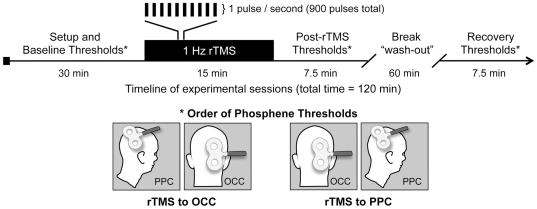
Timeline of experimental sessions in Experiment 2. Each session in Experiment 2 lasted approximately two hours and involved 15 minutes of suppressive 1 Hz repetitive transcranial magnetic stimulation (rTMS) applied to either the pole of the right occipital cortex (OCC) or the right posterior parietal cortex (PPC). At the start of each session, motor and phosphene thresholds were collected to establish baseline excitability. Phosphene thresholds were reassessed immediately following rTMS as well as after a 60-minute break to allow the effects of the rTMS to wear off. Phosphene thresholds were always assessed first at the site that did not receive rTMS, that way a finding of “no change” could not simply be attributed to the effects wearing off.

As illustrated in Figure 6, Experiment 2 consisted of two sessions per participant. Individual sessions were counterbalanced against order bias and separated by at least seven days to minimize the likelihood of carryover effects from the previous session. Both sessions began by assessing the motor threshold followed by baseline threshold values for occipital and parietal phosphenes. Next, 15 minutes of 1 Hz rTMS was applied to one of the two sites, and immediately thereafter, we again assessed the phosphene threshold at both locations. Since 1 Hz rTMS is known to temporarily reduce excitability at the stimulated region [23], [50], [70], [75], we predicted that this manipulation would increase the magnetic field intensity required to elicit phosphenes (i.e., the phosphene threshold) for the site that received rTMS. Furthermore, we hypothesized that any change in thresholds for the site that did not receive rTMS would be evidence of a functional dependency, thus indicating some link or common origin. Following the post-rTMS thresholds, participants took a break for approximately 60 minutes to allow the effects of the rTMS to completely wear off before both phosphenes thresholds were measured for a third and final time. For all time-points, we first assessed the threshold at the site that did not receive rTMS, so that a null change in the threshold measured at the non-rTMS site could not be simply attributed to the effects of rTMS wearing off before we had finished collecting both post-stimulation thresholds.

Data collected for each participant consisted of one motor threshold and three pairs of phosphene thresholds per session. To assess the effect of 1 Hz rTMS, phosphene thresholds were entered into a 2 (rTMS target: occipital, parietal)×2 (phosphene threshold site: occipital, parietal)×3 (time: baseline, post-rTMS, recovery) repeated-measures ANOVA with a 95% confidence interval (α = 0.05). Mauchly's test indicated that the assumption of sphericity had been violated for the main effect of Time, χ^2^(2) = 6.06, *p*<0.05), therefore degrees of freedom were corrected using Huynh-Feldt estimates of sphericity (ε = .83). For all other effects and interactions, the assumption of sphericity was met, all χ^2^'s<5, all *p*'s>0.1, allowing us to use uncorrected degrees of freedom. Post-hoc and planned pair-wise comparisons were computed using *z*-tests (for *n*≥30) and paired-samples *t*-tests (for *n*<30), using a more conservative 99% confidence interval (α = 0.01) to account for multiple comparisons. In addition, we used a Pearson's coefficient (*r*) to examine the correlations between baseline motor and phosphene thresholds, with a 95% confidence interval.
